# The role of HIF-1α in myocardial ischemia protection mechanisms and multi-target rational regulation strategies for natural products

**DOI:** 10.3389/fphar.2026.1823774

**Published:** 2026-08-11

**Authors:** Sisi He, Yuyao Wang, Qi Shi, Yingying Zhang, Xiaofei Wang, Cong Wang, Taiyi Wang

**Affiliations:** 1 Shandong University of Traditional Chinese Medicine, Jinan, Shandong, China; 2 China-Japan Friendship Hospital, Beijing, China

**Keywords:** cell death, HIF-1α, multi-target, myocardial ischemia, natural products

## Abstract

Ischaemic heart disease (IHD) remains a major contributor to global morbidity and mortality from cardiovascular disease. Its core mechanism involves an imbalance in myocardial oxygen metabolism, ultimately leading to cardiomyocyte apoptosis or necrosis. As the central regulator of cellular oxygen sensing and adaptation, hypoxia-inducible factor-1α (HIF-1α) plays a pivotal role in the pathogenesis of IHD by orchestrating key pathological processes—such as angiogenesis, inflammation, apoptosis, and mitochondrial dysfunction—through transcriptional regulation of diverse antioxidant and stress-responsive genes. Despite its importance, therapeutic strategies directly targeting HIF-1α remain limited. In contrast, natural products (NPs) possess a long history of clinical application and demonstrated safety, with distinct advantages including multi-target regulation and low toxicity, making them promising candidates for myocardial protection and drug discovery. Accumulating evidence indicate that NPs act through coordinated, multi-target modulation of the HIF-1α signalling network, though their complex mechanisms require further elucidation. This review summarises current insights into the regulatory role of HIF-1α in myocardial ischaemic injury and highlights the therapeutic potential of NPs in modulating this pathway, providing a foundation for novel anti-ischaemic drug development.

## 1 Background

Cardiovascular diseases (CVDs) remain the leading cause of mortality and chronic disability worldwide, with Ischaemic heart disease (IHD) accounting for the majority of CVD-related deaths (Li Z. et al., 2021). IHD is characterised by myocardial ischaemia and hypoxia resulting from an imbalance between coronary blood flow supply and myocardial oxygen demand, clinically manifesting as angina pectoris, myocardial infarction, or chronic heart failure (Nowbar et al., 2019). Restricted coronary flow triggers myocardial energy metabolism disruption, mitochondrial dysfunction, excessive reactive oxygen species (ROS) generation, and calcium overload, ultimately leading to cardiomyocyte apoptosis or necrosis (Heusch, 2020). The primary treatment for IHD involves revascularisation combined with pharmacotherapy to promptly restore myocardial perfusion (Welsh et al., 2022). However, reperfusion may induce myocardial ischaemia–reperfusion injury (MIRI), involving cell damage, apoptosis, inflammation, and microvascular injury, which worsens prognosis (Hu et al., 2022). Given the severity and complexity of this condition, identifying novel therapeutic targets to mitigate myocardial ischaemic injury is critically important.

Hypoxia-inducible factor (HIF) is a central regulator of cellular adaptation to hypoxia, comprising oxygen-sensitive α subunits (HIF-1α, HIF-2α) and a constitutively expressed β subunit (HIF-1β/ARNT) (Dengler et al., 2014). Hypoxia-inducible factor-1α (HIF-1α) is ubiquitously expressed and responds rapidly to hypoxia. Under normoxia, HIF-1α is hydroxylated and degraded; under hypoxia, this process is inhibited, enabling HIF-1α stabilisation. Stabilised HIF-1α translocates to the nucleus, heterodimerises with HIF-1β, and binds to hypoxia response elements (HREs), initiating adaptive transcriptional programmes (Majmundar et al., 2010).

In recent years, modulation of HIF-1α activity has emerged as a therapeutic target for multiple human diseases. Despite significant progress in the development of drugs targeting HIF-1α, no agents regulating this pathway have yet been applied to the treatment of IHD. Natural products (NPs) have been widely applied for the management of IHD, and modern research has confirmed that some of these compounds can modulate HIF-1α targets (Li et al., 2020). As a core transcription factor in hypoxia adaptation, HIF-1α promotes angiogenesis, enhances glycolysis, and suppresses oxidative stress and apoptosis, thereby protecting cardiomyocytes (Kierans and Taylor, 2021; Mialet-Perez and Belaidi, 2024; Bakleh and Al Haj Zen, 2025). Consequently, NPs have attracted increasing attention in cardiovascular research owing to their high safety, structural diversity, and multi-target properties. A variety of active components such as flavonoids, saponins, and alkaloids have been reported to exhibit cardioprotective effects, making them promising candidates for the development of anti-myocardial ischemic agents (Kumar et al., 2025). Therefore, elucidating HIF-1α-mediated cardioprotective mechanisms of NPs will support the development of novel natural anti-ischaemic drugs.

## 2 Systematic literature search and study selection

To ensure transparency and reproducibility, a systematic literature search was performed in the databases including PubMed and Web of Science. The search strategy combined the following terms: “myocardial ischemia” OR “myocardial infarction” OR “ischaemia-reperfusion injury” AND “HIF-1α” OR “hypoxia-inducible factor-1α” AND “natural product” OR “plant extract” OR “herbal compound”.

Inclusion criteria: (1) *In vitro* or *in vivo* studies investigating cardioprotective effects; (2) Studies clearly reporting HIF-1α-related mechanisms; (3) Full-text articles published in English.

Exclusion criteria: (1) Reviews, meta-analyses, letters, and conference abstracts; (2) Studies without clear HIF-1α functional validation; (3) Studies lacking appropriate experimental controls.

## 3 Role of HIF in ischemia

**FIGURE 1:**
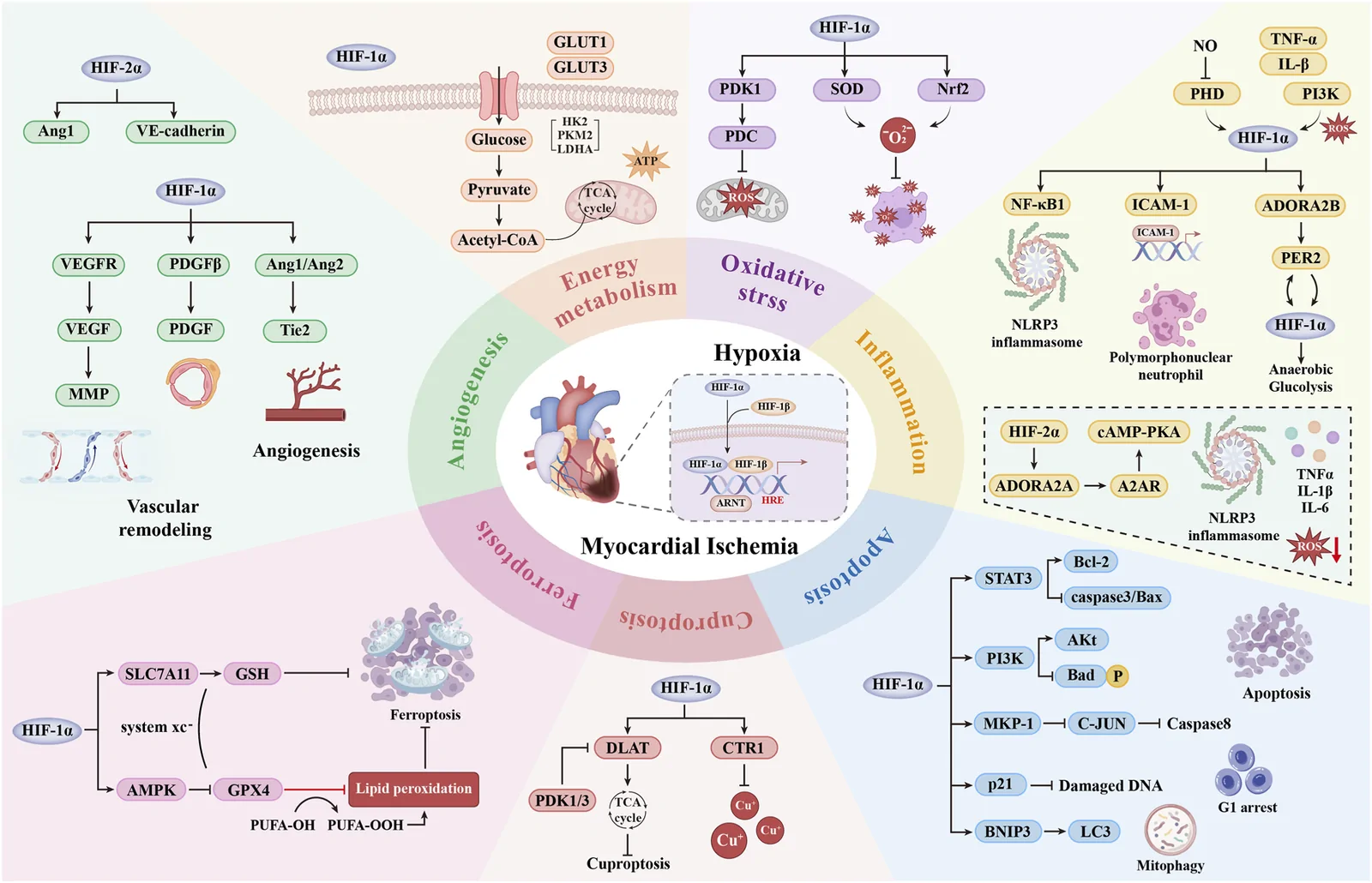
The mechanisms of HIF in treating myocardial ischemia. These mechanisms include: angiogenesis, energy metabolism, oxidative stress, inflammation, apoptosis, ferroptosis and cuproptosis. “↑” denotes activation, stimulation, or promotion, whereas “⊥” denotes inhibition, suppression, or reduction.

Myocardial ischemia is characterized by a hypoxic microenvironment that acts as a central trigger for the stabilization and activation of HIF-1α. Upon activation, HIF-1α initiates a series of interconnected adaptive responses, such as angiogenesis, metabolic reprogramming, the regulation of cardiomyocyte survival, the maintenance of redox homeostasis, and the modulation of programmed cell death. These sequential and interrelated processes work in concert to determine the ultimate fate of cardiomyocytes under ischemic stress (Figure 1).

### 3.1 Regulation of angiogenesis

Angiogenesis is the first-line adaptive response to myocardial ischemia, and HIF-1α functions as the master transcriptional driver. A pivotal discovery by Forsythe et al. (1996) demonstrated that vascular endothelial growth factor (VEGF), a key angiogenic factor, is induced by HIF-1α (Semenza, 2003). HIF markedly upregulates VEGF expression, and secreted VEGF binds to the endothelial cell receptor VEGFR-2, activating the PI3K/AKT and RAS/MAPK pathways to promote endothelial cell survival, proliferation, and migration (Shaw et al., 2024). VEGF induces matrix metalloproteinase (MMP) expression to facilitate endothelial migration and vascular formation (Bergers and Benjamin, 2003).

HIF-1α further enhances angiogenesis by regulating metabolic reprogramming. HIF-1α activation promotes glucose uptake and glycolysis, supporting endothelial cell survival under hypoxic conditions (Madai et al., 2024). Beyond VEGF, other HIF-1α-regulated angiogenesis-related genes also contribute to vascular development. HIF-1α enhances vascular stability by recruiting pericytes to nascent vessels through platelet-derived growth factor (PDGF) upregulation and coordinates vascular remodelling and maturation by regulating the interaction of angiopoietins (Ang-1/Ang-2) with Tie2 receptors, ultimately restoring blood supply to ischaemic tissues. HIF-2α plays a distinct yet complementary role in chronic hypoxia. HIF-2α induces Ang-1 expression and upregulates VE-cadherin to maintain vascular integrity (Park et al., 2016; Xu et al., 2021). Moreover, HIF-2α acts synergistically with HIF-1α to sustain VEGF expression (Park et al., 2016).

In summary, the HIF signalling pathway regulates the entire angiogenic process in ischaemic myocardium through multi-level integration-from acute-phase gene transcription and metabolic reprogramming to chronic-phase vascular structural remodelling. Specifically, HIF-1α not only induces angiogenic factors but also mediates metabolic reprogramming to support cell survival and migration under hypoxia.

### 3.2 Metabolic reprogramming

Metabolic reprogramming constitutes a fundamental adaptive strategy orchestrated by HIF-1α, rather than a passive consequence of hypoxia, to ensure cardiomyocyte survival. Myocardial hypoxia suppresses oxidative phosphorylation, compelling ischaemic cells to switch to anaerobic glycolysis for ATP generation (Lesnefsky et al., 2017). This metabolic shift impairs myocardial cell metabolism and induces cellular injury (Liu et al., 2020). Therefore, maintaining sufficient ATP production through anaerobic glycolysis protects cardiomyocytes from damage and preserves contractile performance (Heusch, 2020). Under anaerobic conditions, sustaining glycolytic flux to ensure ATP availability is crucial for myocardial protection.

Hypoxia signalling serves as a central mechanism in energy metabolism remodelling following myocardial ischaemia, with HIF-1α playing a pivotal regulatory role. HIF-1α enhances glucose uptake by upregulating GLUT1, GLUT3 and hexokinase 2 (HK2), providing substrates for glycolysis. Concurrently, HIF-1α directly activates rate-limiting glycolytic genes and promotes glycolysis via PI3K/Akt-mediated phosphorylation to rapidly generate ATP (Xin et al., 2023). HIF-1α modulates mitochondrial enzyme activity to limit TCA cycle flux, enhance glycolysis and reduce mitochondrial oxygen consumption (Mylonis et al., 2019). Furthermore, HIF-1α suppresses mitochondrial biogenesis by downregulating TFAM and diminishes aerobic respiration (Mialet-Perez and Belaidi, 2024). HIF-1α maintains mitochondrial homeostasis by regulating Drp1, Mfn1 and Mfn2 expression (Xin et al., 2023). HIF-1α-induced miR-210 targets ISCU1/2 to disrupt electron transport chain function and impair oxidative phosphorylation (Chan et al., 2009).

In summary, HIF-1α markedly enhances anaerobic glycolytic activity by directly upregulating glycolysis-related genes and indirectly modulating aerobic metabolic pathways, thereby promoting rapid ATP production and enabling myocardial adaptation to acute hypoxia. Metabolic disorders further trigger oxidative stress and inflammatory activation, both of which are also modulated by HIF-1α.

### 3.3 Immune microenvironment

Inflammation is a double-edged sword in myocardial ischemia, and HIF-1α dynamically shapes the inflammatory response in a time- and context-dependent manner. Myocardial ischaemia and hypoxia induce cardiomyocyte death, triggering an inflammatory response and recruiting immune cells to the ischaemic region for clearance of cellular and extracellular matrix debris (McGettrick and O’Neill, 2020). Macrophage- and neutrophil-derived nitric oxide (NO) stabilises HIF-1α by inhibiting prolyl hydroxylase (PHD) activity and preventing its ubiquitin-mediated degradation. Concurrently, inflammatory cytokines such as tumour necrosis factor-α (TNF-α) and interleukin-1β (IL-1β) promote HIF-1α activation through both ROS-dependent and PI3K-mediated signalling pathways (Brune and Zhou, 2007).

During acute ischaemia, HIF-1α directly binds to the NFKB1 enhancer, promoting NLRP3 inflammasome assembly and stimulating proinflammatory cytokine secretion (Tannahill et al., 2013). In contrast, under prolonged hypoxia, HIF-1α activation reduces chemokine and cell adhesion molecule expression, thereby attenuating inflammatory phenotypes. HIF-1α suppresses transcription of chemokines and ICAM-1, reducing neutrophil retention and dampening inflammation (Madai et al., 2024).

HIF-2α directly upregulates the extracellular nucleotidases CD39 and CD73, significantly increasing adenosine production in hypoxic zones (Ullah et al., 2023). The adenosine receptor ADORA2B, a key damage-associated molecular pattern (DAMP) inhibitor, is a direct transcriptional target of HIF-1α. Activation of ADORA2B stabilises PER2, which interacts with HIF-1α to enhance anaerobic glycolytic capacity during ischaemia (Eltzschig et al., 2013). Conversely, the anti-inflammatory adenosine receptor ADORA2A, a HIF-2α target, is critical for suppressing inflammation *in vivo*. By binding to adenosine A2A receptors (A2ARs) on macrophages, it activates the cAMP–PKA signalling cascade (Requena-Ibáñez et al., 2022). This pathway inhibits macrophage NLRP3 inflammasome activation, proinflammatory cytokine release, and ROS production, reducing inflammatory injury. Additionally, HIF-1α inhibits the TLR4/MyD88/NF-κB and NLRP3 inflammasome pathways to alleviate myocardial inflammation (Chen et al., 2020).

In summary, HIF-1α exerts proinflammatory effects during the acute phase of ischaemia by activating NLRP3 inflammasomes and promoting cytokine secretion. With prolonged hypoxia, it transitions to an anti-inflammatory role by suppressing chemokine and adhesion molecule expression, thereby limiting inflammatory infiltration. HIF-2α primarily inhibits NLRP3 activation and ROS production via the adenosine–A2A/cAMP–PKA pathway, enhancing anti-inflammatory signalling (Requena-Ibáñez et al., 2022). Although HIF-1α and HIF-2α both serve as key regulators of hypoxic signaling, they exhibit markedly distinct expression patterns and functional roles during myocardial ischemia. HIF-1α is mainly activated in the acute phase of ischemia, exerting rapid and transient effects to enhance glycolysis, maintain energy metabolism, and promote cardiomyocyte survival (Heck-Swain and Koeppen, 2023). In contrast, HIF-2α is induced relatively slowly and functions predominantly in the subacute or chronic stage, mainly participating in sustained angiogenesis, vascular remodeling, and long-term tissue repair (Knutson et al., 2021). These two isoforms display clear spatiotemporal differences and complementary functions rather than overlapping effects, which jointly determine the adaptive outcome of the heart under ischemic stress. In summary, Both HIF-1α and HIF-2α complement each other temporally and mechanistically to jointly regulate inflammatory balance during ischemia. Inflammation and metabolism both contribute to ROS overproduction, a key trigger of oxidative stress.

### 3.4 Oxidative stress

Oxidative stress is a common downstream pathological event linking metabolism, inflammation, and cell death in myocardial ischemia, and HIF-1α serves as a critical redox regulator. HIF-1α reduces mitochondrial ROS production by suppressing oxidative metabolism and inhibits inflammation-driven ROS generation. Oxidative stress arises when the production of ROS exceeds the capacity of endogenous antioxidant systems, thereby disrupting redox homeostasis and inducing cellular injury (Batty et al., 2022). Mitochondria, the primary source of ROS, experience electron transport chain disruption under hypoxic conditions, resulting in elevated ROS generation. These ROS induce oxidative damage to proteins, DNA, and lipids, compromising cellular viability (Ham and Raju, 2017). Progressive ROS accumulation disrupts the proton gradient, leading to mitochondrial membrane rupture and apoptotic factor release, ultimately resulting in cell death (Bock and Tait, 2020).

Interestingly, under hypoxia, mitochondrial-derived ROS also contribute to the stabilisation of HIF-1α (Cadenas, 2018). Once activated, HIF-1α initiates a multifaceted adaptive programme to mitigate oxidative stress and restore redox balance. It transcriptionally regulates mitochondria-associated genes to enhance mitochondrial function and facilitate adaptation to hypoxic stress (Chowdhury et al., 2018). A key metabolic adjustment is the induction of PDH kinase 1 (PDK1), which inhibits the tricarboxylic acid (TCA) cycle and diverts glucose metabolites towards glycolysis, thereby reducing mitochondrial respiration and limiting ROS production (Pan et al., 2024). Concurrently, HIF-1α suppresses oxidative metabolism by downregulating mitochondrial biogenesis (Wang et al., 2022).

In addition, HIF-1α strengthens the cellular antioxidant defence system by upregulating the Nrf2-mediated response, resulting in increased levels of glutathione and superoxide dismutase 2 (SOD2), which collectively contribute to redox homeostasis and cytoprotection under hypoxic conditions. Excessive oxidative stress and unresolved inflammation ultimately lead to regulated cell death, which is also tightly controlled by HIF-1α.

### 3.5 Cell survival and death regulation

As the final executor of ischemic injury, cardiomyocyte death is precisely modulated by HIF-1α, which acts as the upstream regulator that determines cardiomyocyte survival or death under ischemic stress. Myocardial ischaemia triggers direct hypoxic damage and ischaemia–reperfusion injury. Sustained ischaemia leads to irreversible cardiomyocyte loss (Wu et al., 2018). Hypoxia from inadequate coronary perfusion constitutes the pathophysiological basis of IHD (Datta Chaudhuri et al., 2020). As terminally differentiated cells with limited proliferative capacity, cardiomyocytes cannot be efficiently regenerated following injury, impeding functional recovery. Promoting cardiomyocyte survival has emerged as a critical research priority in myocardial ischaemia (Laflamme and Murry, 2011).

#### 3.5.1 Apoptosis

HIF-1α functions upstream to inhibit apoptosis and promote cardiomyocyte survival. During moderate hypoxia, HIF-1α forms heterodimers with ARNT to activate transcriptional programmes that exert anti-apoptotic effects (Piret et al., 2002). Within the hypoxic–ischaemic microenvironment, HIF-1α acts as a central regulator, inhibiting apoptosis through multiple complementary mechanisms.

Activated HIF-1α induces STAT3 expression, upregulates the anti-apoptotic protein Bcl-2, and downregulates the pro-apoptotic proteins cleaved caspase-3 and Bax, thereby reducing oxidative stress and myocardial cell apoptosis (Yang et al., 2021). Additionally, HIF-1α enhances MKP-1 activity, which dephosphorylates JNK and suppresses c-Jun transcriptional activity. This downregulates Fas ligand expression and inhibits the Fas-mediated caspase-8 activation pathway, blocking death receptor-induced apoptosis (Liu et al., 2021).

HIF-1α induces p21 expression to arrest the cell cycle at the G1 phase, preventing apoptosis triggered by DNA damage under hypoxic conditions. It activates the PI3K/Akt pathway to phosphorylate and inhibit the pro-apoptotic protein Bad, enhancing cellular survival capacity (Zhang et al., 2018). Mortalin directly interacts with HIF-1α and inhibits apoptosis by preventing Bax recruitment to the mitochondria (Yfantis et al., 2023). Moreover, HIF-1α induces BNIP3-mediated mitophagy by binding to LC3 to promote autophagosome formation and disrupt mitochondrial membrane potential, facilitating the clearance of damaged mitochondria and reducing pro-apoptotic factor release (Gustafsson and Gottlieb, 2009).

Collectively, these mechanisms form a multilayered regulatory network through which HIF-1α protects cardiomyocytes from apoptosis under hypoxic conditions, thereby promoting myocardial survival and adaptation during ischaemic stress.

#### 3.5.2 Ferroptosis

HIF-1α acts upstream to regulate iron metabolism and lipid peroxidation, thus controlling ferroptosis. Ferroptosis is a non-apoptotic form of regulated cell death characterised by its dependence on iron and initiation through lipid peroxidation (Farmer and Mueller, 2013; Bogdan et al., 2016). It involves the accumulation of ROS and lipid peroxides within cells, leading to disruption of membrane structure and loss of cellular integrity (Dixon et al., 2012; Jiang et al., 2021). Ferric iron (Fe^3+^) transported via transferrin receptor (TFRC) is reduced to ferrous iron (Fe^2+^) and released into the cytoplasm. The Fenton reaction generates hydroxyl radicals that activate lipoxygenases and induce lipid peroxidation (Porter et al., 1995). Erastin blocks system Xc^−^, causing GSH depletion and GPX4 inactivation. Lipoxygenases and non-enzymatic reactions oxidize PUFA-PLs, triggering ferroptotic cell death. The core mechanisms involve lipid peroxidation, GSH dysregulation, and iron imbalance (Yang et al., 2014; Wu et al., 2021).

During I/R injury, oxidative bursts and elevated iron contribute to ferroptosis. Ferroptosis represents a potential therapeutic target in myocardial I/R injury and heart failure (Wu et al., 2021). In cardiomyocytes, miR-210-3p, which is enriched in exosomes derived from cardiac microvascular endothelial cells under hypoxic conditions (1% O_2_), attenuates erastin-induced ferroptosis by directly suppressing TFRC mRNA expression (Lei et al., 2022). Notably, the role of HIF-1α in ferroptosis is tightly regulated by the ischemic context. In acute myocardial ischaemia, moderate HIF-1α activation exerts anti-ferroptotic effects, whereas in prolonged ischaemia, excessive HIF-1α activation aggravates iron overload and lipid peroxidation by upregulating TFRC and suppressing ferritin, thereby promoting ferroptosis.

Thus, the interaction between HIF and ferroptosis represents a dynamic equilibrium in ischaemic disease. Precise regulation of HIF activity and its downstream pathways may enable the dual objectives of protecting viable cells and eliminating damaged ones.

#### 3.5.3 Cuprotosis

HIF-1α serves as an upstream regulator of copper transport and mitochondrial metabolism to affect cuprotosis. Cuprotosis is a recently identified, non-apoptotic form of regulated cell death triggered by excessive copper accumulation. It occurs through the aggregation of acylated mitochondrial proteins, leading to disruption of mitochondrial metabolism (Cobine and Brady, 2022). Copper enters the mitochondrial matrix via family 25 member 3 (SLC25A3), and ferredoxin-1(FDX1) reduces Cu^2+^ to Cu^+^. Cu^+^ binds to thioacylated TCA cycle enzymes, inducing protein aggregation and impairing mitochondrial function (Cobine and Brady, 2022).

During ischaemia, cells shift to glycolysis for energy production, reducing mitochondrial respiration and potentially diminishing their susceptibility to copper-induced death (He and James Kang, 2013). In ischaemia–reperfusion models, the acute depletion of copper may represent a stress-adaptive response that limits mitochondrial copper overload, thereby supporting cell survival (Tsvetkov et al., 2022). Conversely, copper supplementation can restore HIF-1α activity and promote angiogenesis, as shown in a hypertrophic cardiomyopathy mouse model, but may simultaneously increase the risk of copper-induced cell death (Jiang et al., 2007).

Therefore, in the treatment of IHD, it is essential to maintain a delicate balance between the pro-angiogenic benefits of copper supplementation and the potential cytotoxicity associated with cuprotosis.

## 4 Natural products

Derived from plants and microorganisms, NPs offer unique advantages, including structural diversity, potent bioactivity, multi-target effects, and low toxicity (Atanasov et al., 2021). These attributes make NPs a major focus in novel drug discovery and myocardial protection strategies. Structurally, NPs can be classified into flavonoids, saponins, phenolic acids, alkaloids, terpenoids, and other categories. Extensive research demonstrates that numerous NPs exert cardioprotective effects through diverse mechanisms, including attenuation of oxidative stress (Zhang et al., 2022), inhibition of inflammation (Li et al., 2025), regulation of apoptosis (Cheng et al., 2024), and improvement of energy metabolism (Su et al., 2022). In particular, latest evidence has highlighted the promising roles of NPs in targeting complex signaling networks associated with myocardial ischemia injury (Liu et al., 2025; Yao et al., 2025; Yue et al., 2025). This multi-target pharmacological profile makes NPs particularly well suited to address the complex and multifactorial pathophysiology of IHD (Table 1). Chemical structures of all monomeric compounds are displayed in Supplementary Figure S1.

**TABLE 1:** Experimental model and conclusion of natural products improving myocardial ischemic injury.

Natural product	Dose	Model	Route	Direct target	HIF-1α regulation	Upstream/Downstream signaling	Core pharmacological effect	Core mechanism
Carthamus tinctorius L. (Asteraceae)	1.25, 5,20 μmol/L and 20 mg/kg; 5, 10, 20 μM and 40 mg/kg	CMECs and SD rats; H9c2 cells and C57BL/6J mice	Cell culture and orally	HIF-1α	↑	HIF-1α/VEGFA/Notch1; HIF-1α/SLC7A11/GPX4	Promote angiogenesis; inhibit ferroptosis; alleviate I/R injury	Stabilize HIF-1α to activate angiogenic and anti-ferroptosis pathways
Moschus moschiferus L. (Moschidae)	2 mg/kg	C57BL/6J mice	Orally	HIF-1α	↑	HIF-1α/VEGFA	Enhance collateral angiogenesis; improve perfusion	Upregulate HIF-1α/VEGFA to promote neovascularization
Apigenin	1, 3, 10 μM,	H9c2 cells	Cell culture	HIF-1α	↓	HIF-1α/PPARα/CPT-1	Improve glucolipid metabolic disorder	Inhibit excessive HIF-1α to normalize metabolic reprogramming
Crataegus pinnatifida Bunge (Rosaceae)	100 mg/kg	SD rats	Orally	HIF-1α	↑	Akt/HIF-1α	Inhibit apoptosis; reduce I/R injury	Activate Akt to upregulate HIF-1α and suppress apoptosis
Dalbergia odorifera T.C.Chen (Fabaceae)	25, 50, 100 mg/kg	SD rats	Orally	HIF-1α	↑	HIF-1α/NF-κB/IL-6	Alleviate inflammatory injury	Activate HIF-1α to inhibit NF-κB-mediated inflammation
Sophora japonica L. (Fabaceae)	10 μM	H9c2 cells	Cell culture	HIF-1α	↑	PI3K/Akt/HIF-1α	Alleviate oxidative stress and inflammation; reduce OGD/R injury	Activate PI3K/Akt/HIF-1α axis to suppress oxidative stress and inflammation
Astragalus membranaceus (Fisch.) Bunge (Fabaceae)	10 mg/kg	C57BL/6 mice	Orally	HIF-1α	↑	miR-411/HIF-1α; Notch1	Promote angiogenesis; improve cardiac function	Stabilize HIF-1α via miR-411 to enhance vascular repair
Rhodiola rosea L. (Crassulaceae)	10, 100, 250, 500, 750, 1000 nM	H9c2 cells	Cell culture	EGLN1	↑	EGLN1/HIF-1α	Inhibit apoptosis; improve mitochondrial metabolism	Inhibit EGLN1 to upregulate HIF-1α and enhance survival
Panax notoginseng (Burkill) F.H.Chen (Araliaceae)	50 mM	SD rats	Orally	HIF-1α	↑	HIF-1α/BNIP3	Enhance mitophagy; alleviate I/R injury	Activate HIF-1α/BNIP3 to clear damaged mitochondria
Panax ginseng C.A.Mey. (Araliaceae)	35 mg/kg; 50, 100, 150, 200 μM	Diabetic rats; H9c2 cells	Orally; Cell culture	HIF-1α	↑	HIF-1α-ERK; PI3K/Akt/HIF-1α/VEGF	Promote angiogenesis; alleviate I/R injury	Activate PI3K/Akt via steroid hormone-like structure to upregulate HIF-1α/VEGF
Ferulic acid	50 μM	HUVECs	Cell culture	HIF-1α	↑	HIF-1α/VEGF/PDGF	Promote angiogenesis	Upregulate HIF-1α to induce angiogenic factors
Salvia miltiorrhiza Bunge (Lamiaceae)	25 mg/kg; 50 μM	C57BL/6 mice; CMECs	Orally/Cell culture	HIF-1α	↑	HIF-1α/VEGF	Enhance endothelial proliferation	Inhibit ubiquitination to stabilize HIF-1α
Coptis chinensis Franch. (Ranunculaceae)	300 mg/kg; 50 μM	SD rats; H9c2 cells	Orally	HIF-1α	↑	HIF-1α/BNIP3	Inhibit apoptosis; protect against I/R injury	Induce mitophagy via HIF-1α/BNIP3
*Centella asiatica* (L.) Urb. (Apiaceae)	0.1, 1, 10, 100 μM	H9c2 cells	Cell culture	Akt/GSK-3β	↑	Akt/GSK-3β/HIF-1α	Inhibit apoptosis; improve energy metabolism	Activate Akt/GSK-3β to upregulate HIF-1α

### 4.1 Flavonoids

According to the IUPAC nomenclature recommendations for flavonoids (Abbate et al., 2018), Hydroxysafflor yellow A is structurally classified as a quinochalcone due to its open-chain C6-C3-C6 skeleton, rather than a typical flavonoid with a closed heterocyclic ring. Therefore, we have updated its classification in Table 1.

Flavonoids are the most extensively studied and well-evidenced class of natural products targeting HIF-1α for myocardial ischemia. Most flavonoids exert protective effects by activating HIF-1α, whereas a few inhibit abnormally elevated HIF-1α, thereby exhibiting precise differential regulation. Flavonoids belong to a major group of polyphenolic compounds characterized by a conserved C6-C3-C6 diphenylpropane skeleton, and numerous flavonoid monomers have been verified to regulate HIF-1α in myocardial ischemia models.

Hydroxysafflor yellow A (HSYA, PubChem CID: 5315016), a typical chalcone flavonoid glycoside isolated from *Carthamus tinctorius* L., contains a polyhydroxylated chalcone core with multiple glycosyl substituents. Chemical structure: Supplementary Figure S1A. As a widely recognized traditional cardiovascular herb, Carthamus tinctorius L. has long been used in Asian populations; It is recognised for improving blood circulation and eliminating blood stasis, and thus plays a significant role in the management of cardio-cerebrovascular diseases (Zhao et al., 2020). HSYA acts as an upstream regulator to stabilize and upregulate HIF-1α, and simultaneously mediates two critical cardioprotective pathways: it promotes the migration of myocardial microvascular endothelial cells and angiogenesis through the HIF-1α/VEGFA/Notch1 axis (Ge et al., 2024), and inhibiting ferroptosis via the HIF-1α/SLC7A11/GPX4 axis (Ge et al., 2023), thereby achieving dual protection of “pro-angiogenesis and anti-ferroptosis” and alleviating myocardial ischemia/reperfusion (I/R) injury.

Muscone (PubChem CID: 7160826) from *Moschus moschiferus* L. Chemical structure: Supplementary Figure S1B. *Moschus moschiferus* L. has been traditionally used for resuscitation and relieving angina; it is characterised by low cytotoxicity and multiple pharmacological activities, including anti-tumour, anti-dementia, and anti-cerebral ischaemia effects (Wang et al., 2020). Muscone upregulates HIF-1α and VEGFA, thereby promoting collateral circulation in the ischemic infarct region (Du et al., 2018).

Apigenin (PubChem CID: 5280443) Chemical structure: Supplementary Figure S1C. Apigenin is widely distributed in common vegetables and medicinal herbs such as celery and chamomile, which exhibits therapeutic potential against a range of chronic diseases, including cancer, Alzheimer’s disease, depression, diabetes, and cardiovascular disorders (Thomas et al., 2023). Apigenin acts differently from most flavonoids by inhibiting excessive HIF-1α activation, improving glycolipid metabolic disorders induced by hypoxia or angiotensin II, and downregulating abnormal glycolysis and lipid synthesis. This regulatory pattern represents a rational strategy to correct pathological HIF-1α activation (Zhu et al., 2019).

The Vitexin from *Crataegus pinnatifida* Bunge (Rosaceae), mainly including vitexin, rutin and hyperoside Chemical structures: Supplementary Figure S1D. Crataegus pinnatifida Bunge i is widely consumed as both food and medicine; its fruits are orally administered in the general population and clinical practice to dilate coronary vessels, reduce blood lipid levels, and relieve angina pectoris (Lu et al., 2023). This Vitexin attenuates cardiomyocyte apoptosis via regulating the Akt/HIF-1α pathway (Jayachandran et al., 2010).

Latifolin (PubChem CID: 1548466) Chemical structujre: Supplementary Figure S1E. Latifolin isolated from *Dalbergia odorifera* T.C.Chen, which is traditionally used to activate blood circulation and alleviate pain; It exhibits anti-ageing, anti-carcinogenic, anti-inflammatory, and cardioprotective effects (Yun et al., 2022). Latifolin mainly upregulates HIF-1α to block the HIF-1α/NF-κB/IL-6 inflammatory pathway, thereby alleviating inflammatory injury in myocardial infarction (Lai et al., 2020).

Troxerutin (PubChem CID: 10816) Chemical structure: Supplementary Figure S1F. Derived from *Sophora japonica* L. Sophora japonica L., which has long been used to improve vascular function; its flower buds are orally taken in folk and clinical practice to alleviate hypertension, coronary heart disease and myocardial ischemic injury (Najafi et al., 2018). Troxerutin attenuates oxygen-glucose deprivation and reoxygenation-induced oxidative stress and inflammation by enhancing the PI3K/AKT/HIF-1α signaling pathway in H9C2 cardiomyocytes (Yu et al., 2020).

Comparison within the class: Flavonoids do not show a single pattern of HIF-1α activation. HSYA from *C. tinctorius* L. and muscone from *M. moschiferus* L. mainly promote angiogenesis and inhibit ferroptosis; apigenin mainly inhibits excessive HIF-1α activation and corrects metabolic disorders; flavonoids from *C. pinnatifida* Bunge, *D. odorifera* T.C.Chen and *S. japonica* L. mainly exert anti-apoptotic and anti-inflammatory effects, with highly differentiated targets and pharmacological effects, reflecting multi-dimensional regulatory characteristics.

### 4.2 Saponins

All saponins consistently upregulate HIF-1α but differ significantly in downstream pathways and functional emphases, representing a typical class of rational multi-target regulation. Saponins are amphiphilic components composed of a triterpenoid or steroidal aglycone and one or more sugar chains, and representative monomeric compounds exert distinct cardioprotective effects via the HIF-1α signalling pathway.

Astragaloside IV (PubChem CID: 127931) Chemical structure: Supplementary Figure S1G. Isolated from *Astragalus membranaceus* (Fisch.) Bunge (Fabaceae), one of the most commonly used cardiotonic herbs; its root is widely orally consumed by the general public and patients to enhance cardiac function and relieve myocardial fatigue and ischemic injury (Tan et al., 2020). Astragaloside IV stabilizes HIF-1α upstream through the miR-411 axis, significantly promotes angiogenesis, improves cardiac function after myocardial infarction, and focuses on blood supply reconstruction and repair of ischemic tissues (Yang et al., 2024).

Salidroside (PubChem CID: 637014) Chemical structure: Supplementary Figure S1H. Derived from *Rhodiola rosea L. (*Crassulaceae*)*, which has long been used in high-altitude regions to enhance hypoxia tolerance; it is orally administered to resist hypoxic stress, maintain cardiac metabolism and alleviate ischemia-related discomforts (Han et al., 2022). Salidroside directly targets EGLN1, an upstream regulator of HIF-1α, to upregulate HIF-1α expression, inhibits apoptosis of hypoxic cardiomyocytes, improves mitochondrial energy metabolism, and focuses more on maintaining cell survival and energy homeostasis (Zhang et al., 2024).

Notoginsenoside R1 (PubChem CID: 14225303) Chemical structure: Supplementary Figure S1I. Isolated from *Panax notoginseng (Burkill) F.H.Chen (Araliaceae)*, a classic herb for activating blood circulation and resolving stasis; its root is widely orally used in clinical and folk medicine to relieve chest pain, myocardial infarction and ischemic heart disease (Xu et al., 2019). Notoginsenoside R1 specifically activate the HIF-1α/BNIP3 pathway to strongly induce mitophagy and eliminate damaged mitochondria after ischemic injury (Liu et al., 2019). Its regulatory effect on mitophagy is significantly stronger than that on angiogenesis.

Ginsenoside Rg1 (PubChem CID: 441928) Chemical structure: Supplementary Figure S1J. Derived from Panax ginseng C.A.Mey. *(Araliaceae)*, Panax ginseng C.A.Mey. a globally recognized cardioprotective tonic; its root is orally taken to strengthen cardiac function and improve myocardial tolerance to ischemia and hypoxia (Han et al., 2024). Ginsenoside Rg1 upregulate HIF-1α expression by activating the PI3K/AKT signaling pathway, inhibits cardiomyocyte apoptosis under hypoxic conditions, promotes the proliferation of vascular endothelial cells, and focuses on enhancing the tolerance of cardiomyocytes to ischemic damage and promoting vascular repair (Chengjun Yuan et al., 2019).

Comparison within the class: All saponins activate HIF-1α but have clear functional preferences: astragaloside IV from *A. membranaceus* (Fisch.) Bunge (Fabaceae) tends to promote angiogenesis; salidroside from *R. rosea* L. (Crassulaceae) tends to regulate metabolism and exert anti-apoptotic effects; saponins from *P. notoginseng* (Burkill) F.H.Chen (Araliaceae) tend to induce mitophagy; ginsenosides from *Panax ginseng* C.A.Mey. (Araliaceae) tend to enhance cardiomyocyte tolerance and vascular repair. They form a synergistic regulatory pattern of “same pathway, different branches”.

### 4.3 Phenolic acids

Phenolic acids mainly exert protective effects by enhancing the stability of HIF-1α, with targets more concentrated on vascular endothelium and angiogenesis. Phenolic acids are aromatic compounds characterized by a C6-C1 or C6-C3 phenolic skeleton, and their typical monomeric components exert anti-ischemic effects by stably regulating HIF-1α activity.

Ferulic acid (PubChem CID: 445858) Chemical structure: Supplementary Figure S1K. Ferulic acid is abundant in Angelica sinensis, Ligusticum chuanxiong, and cereal crops, which have long been utilized in traditional medicine to promote blood circulation, dissipate blood stasis, and protect cardiac function in clinical and folk populations (Li D. et al., 2021). Ferulic acid, a classic hydroxycinnamic acid monomer, upregulates HIF-1α, thereby promoting the expression of angiogenesis-related factors such as VEGF and PDGF, and shows significant pro-angiogenic effects *in vitro* and *in vivo* (Lin et al., 2010).

Salvianic acid A (Danshensu, PubChem CID: 632467) Chemical structure: Supplementary Figure S1L. Isolated from *Salvia miltiorrhiza Bunge (Lamiaceae)*, the most representative herb for cardiovascular diseases; its root is widely orally used in clinics and folk populations to improve myocardial microcirculation and attenuate I/R injury (Song et al., 2019). Salvianic acid A inhibits the ubiquitination and degradation of HIF-1α to prolong its half-life, continuously activates the HIF-1α/VEGF pathway, and more potently promotes the proliferation of myocardial microvascular endothelial cells (Liu et al., 2024).

Comparison within the class: Both compounds center on HIF-1α-mediated angiogenesis but act at different regulatory steps: ferulic acid mainly promotes HIF-1α expression; Ferulic acid mainly reduces HIF-1α degradation, both ultimately achieving stable activation of the HIF-1α pathway.

### 4.4 Alkaloids

Represented by berberine, alkaloids have a highly concentrated regulatory mode, mainly regulating mitophagy and apoptosis via HIF-1α. Alkaloids are nitrogen-containing heterocyclic compounds with potent cardiovascular activities, and their representative monomeric components precisely modulate mitochondrial quality control through HIF-1α signaling.

Berberine (PubChem CID: 2353) Chemical structure. Isolated from *Coptis chinensis* Franch. (Ranunculaceae), which has long been used for its anti-inflammatory and detoxifying activities; its rhizome is orally administered in clinical practice to attenuate inflammation-related myocardial injury and I/R damage (Fang et al., 2022). Berberine activates the HIF-1α/BNIP3 pathway upstream to trigger mitophagy, inhibits cardiomyocyte apoptosis induced by ischemia/reperfusion, reduces myocardial infarct size and reperfusion injury, and exerts prominent protective effects on mitochondrial homeostasis (Zhu et al., 2020).

### 4.5 Terpenoids

Terpenoids do not act directly on the HIF-1α protein, but regulate HIF-1α indirectly through upstream kinases or non-coding RNAs, representing typical upstream multi-level regulation. Terpenoids are lipid components composed of isoprene units, and their representative monomers modulate HIF-1α activity through upstream kinase cascades.

Asiatic acid (PubChem CID: 101686) Chemical structure. *Centella asiatica* (L.) Urb. which is traditionally used to improve microcirculation; it is orally administered in folk use to protect endothelial function and alleviate hypoxic myocardial injury (Qiu et al., 2022). Asiatic acid Isolated from *C. asiatica (L.) Urb. (Apiaceae)*, activates HIF-1α upstream through the Akt/GSK-3β signaling pathway, improves energy metabolism, inhibits hypoxia/reoxygenation-induced apoptosis, and maintains mitochondrial function (Huang et al., 2016).

## 5 Discussion

**FIGURE 2:**
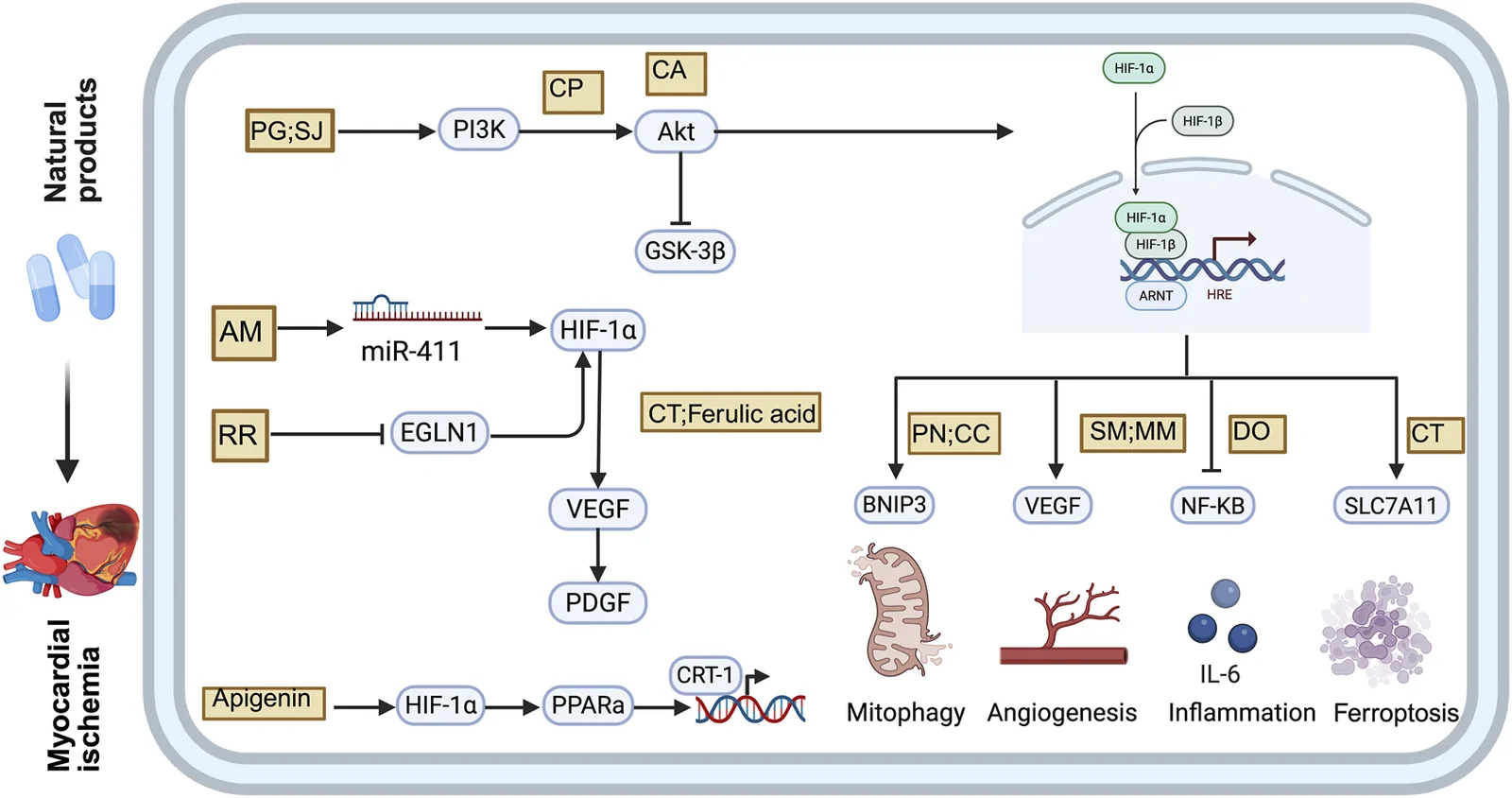
Natural products target HIF-1α to alleviate myocardial ischemia. CT: Carthamus tinctorius L, MM: Moschus moschiferus L, Apigenin: Apigenin, CP: Crataegus pinnatifida Bunge, DO: Dalbergia odorifera T.C.Chen, SJ: Sophora japonica L, AM: Astragalus membranaceus (Fisch.) Bunge, RR: Rhodiola rosea L, PN: Panax notoginseng (Burkill) F.H.Chen, PG: Panax ginseng C.A.Mey, Ferulic acid: Ferulic acid, SM: Salvia miltiorrhiza Bunge, CC: Coptis chinensis Franch, CA: *Centella asiatica* (L.) Urb. “↑” denotes activation, stimulation, or promotion, whereas “⊥” denotes inhibition, suppression, or reduction. During the acute phase of myocardial ischaemia, cardiac tissue is subjected to severe hypoxia and rapid energy depletion. Under such conditions, moderate activation of HIF-1α acts as a central regulatory node that initiates adaptive cardioprotective responses, including enhanced glycolytic metabolism to sustain ATP production, promoted angiogenesis to improve tissue perfusion, and mediated anti-inflammatory and anti-apoptotic effects, all of which constitute a vital endogenous survival mechanism for cardiomyocytes. Compounds such as *Carthamus* tinctorius L (Asteraceae), *Astragalus membranaceus* (Fisch.) Bunge (Fabaceae) and *Panax notoginseng (Burkill)* F.H.Chen (Araliaceae) exert effective myocardial protection by upregulating HIF-1α expression and reinforcing these endogenous defensive pathways. By contrast, under chronic hypoxia or metabolic disturbance, sustained and excessive activation of HIF-1α shifts toward maladaptive and pathological consequences. Prolonged dysregulated signalling contributes to glucolipid metabolic imbalance, lipid accumulation, excessive inflammatory responses, and pathological myocardial remodelling, thereby exacerbating myocardial injury. In such settings, inhibition of overactivated HIF-1α is essential to restore metabolic homeostasis. Apigenin serves as a representative agent that attenuates hyperactive HIF-1α signalling, ameliorates glucolipid metabolic disorders, and consequently confers cardioprotective effects.

One of the leading causes of mortality worldwide, IHD poses a major global public health challenge. Therefore, developing effective therapeutic approaches to control IHD progression, delay disease advancement, and alleviate symptoms is of paramount importance. This study summarises the mechanisms through which NPs target HIF-1α in the treatment of ischaemia, systematically compiling evidence on how various NPs modulate HIF-1α in cellular and animal models. The findings provide a theoretical basis for identifying anti-ischaemic drug candidates targeting HIF-1 and exploring potential lead compounds from natural sources. Among these compounds, flavonoid and saponin demonstrate the most potent anti-ischaemic activity. They alleviate ischaemic injury through multiple mechanisms by modulating HIF-1α across diverse molecular and cellular dimensions, including inhibition of apoptosis, promotion of angiogenesis, suppression of inflammation, regulation of energy metabolism, and attenuation of ferroptosis and cuprotosis (Figure 2).

Myocardial injury caused by ischaemia is a multifactorial process involving numerous physiological and pathological pathways. Therapies targeting a single pathway often show limited efficacy, suggesting that coordinated multi-target modulation of the HIF network may represent a more effective and reliable treatment strategy. Notably, this multi-target strategy does not simply mean natural products have multiple components. It refers to a rational, sequential, and context-dependent intervention that simultaneously targets the upstream regulators, core transcription factor HIF-1α, and its downstream signaling networks. By selectively activating or inhibiting HIF-1α according to the ischemic stage, and coordinately regulating angiogenesis, metabolism, inflammation, and cell death, natural products achieve integrated and precise cardioprotection. Multi-target coordination refers to the synergistic and complementary actions of multiple targets that collectively address different aspects of disease pathogenesis to achieve superior therapeutic outcomes. Target selection, however, must be based on defined upstream–downstream relationships within key signalling pathways. Within the HIF pathway, HIF and its regulatory factors exist in a hierarchical relationship. Under hypoxic conditions, HIF activation triggers the expression of a broad array of downstream genes. The treatment of IHD primarily involves four key processes: angiogenesis, inflammation control, energy metabolism, and programmed cell death. Each of these mechanisms involves multiple HIF-related targets and interactions with other signalling cascades.

For instance, in myocardial ischaemia, HIF-1α not only promotes endothelial cell survival, proliferation, and migration via VEGF signalling but also interacts with the TGF-β1/Smad3 pathway to induce MMP expression. This degrades the extracellular matrix, guiding endothelial cell migration and vascular bud formation. Hydroxycrocetin activates HIF-1α while regulating VEGF downstream signalling via Notch1, thereby promoting angiogenesis (Ge et al., 2024). Similarly, muscone acts on the same pathway to promote the formation of stable collateral circulation in the ischaemic border zone (Du et al., 2018). These multidimensional interactions highlight how HIF cooperates with other molecular targets to achieve superior therapeutic outcomes compared to single-pathway modulation.

NPs thus offer effective strategies against myocardial ischaemia through multi-target coordination of the HIF pathway and its interacting networks. Multi-component formulations enhance both the breadth and precision of cardioprotection by simultaneously regulating multiple pathways involved in cell function and metabolism. Compared with single-compound drugs, multi-component therapies show distinct advantages: their diverse constituents act on multiple molecular targets within the ischaemic myocardium, conferring integrated and robust protection (Li et al., 2024). Studies have shown that combination therapies provide superior protection against MIRI compared with monotherapy (Souri et al., 2023). These findings underscore the feasibility and superiority of a multi-target, multi-phase, and multi-component integrated strategy for managing myocardial ischaemia.

Despite promising preclinical evidence, translating HIF-targeted strategies into successful clinical therapies remains challenging. HIF stabilisers are among the few drugs developed for myocardial ischaemia, yet their application is limited by multisystemic side effects and cardiovascular risks due to non-specific HIF activation. In contrast, modern combination regimens—such as the “new quadruple therapy” for heart failure (ARNI + β-blocker + MRA + SGLT2 inhibitor + vericiguat)—use a multi-pathway, early-initiation, low-dose approach. This strategy overcomes the limitations of traditional stepwise medication escalation, resulting in improved survival and quality of life in heart failure patients (Van Essen et al., 2025).

Moreover, evidence suggests that HIF functions as a “synergistic signalling hub” integrating multiple interventions. Post-ischaemic conditioning (IPoC) upregulates HIF-1α, activating AMPK and RISK pathways to reduce myocardial reperfusion injury and improve cardiac function (Yang et al., 2023). Pharmacological modulation of HIF-1α can also act synergistically with other molecular targets, such as metabolic or angiogenic pathways. Long non-coding RNA (lncRNA) TUG1 suppresses the HIF-1α/VEGF axis after myocardial infarction, while HIF-1α overexpression promotes angiogenesis. Remote ischaemic preconditioning counteracts this inhibitory effect by negating TUG1-mediated suppression of the HIF-1α/VEGF pathway, thereby restoring angiogenic activity (Dang et al., 2023).

Mitochondria-targeted HIF regulation represents another promising therapeutic avenue. Mitochondrial dysfunction is a central feature of ischaemic cardiomyopathy, and HIF-1α supports ATP synthesis and cell survival by regulating mitochondrial metabolism (Mialet-Perez and Belaidi, 2024). Simultaneous targeting of HIF-1α and mitochondrial quality control pathways may enhance protection, as evidenced by the mitigation of myocardial infarction injury and improvement of cardiac function through the PHD3/HIF-1α pathway.

In summary, simultaneous regulation of HIF and its associated pathways represents a promising therapeutic strategy for complex cardiovascular conditions such as ischaemic cardiomyopathy, myocardial infarction, and reperfusion injury. Multi-drug combinations enable synergistic, multi-target interventions that offer stronger protection than monotherapies. Future research should focus on optimising rational synergistic combinations of compounds—including NPs—targeting HIF and related pathways to maximise clinical efficacy.

## 6 Limitations and future research priorities

Despite the promising cardioprotective effects of natural products mediated through HIF-1α signalling, several critical limitations in the current evidence base must be acknowledged. Most available studies remain at the preclinical stage, with a lack of clinical evidence in human subjects. Dose–response relationships, long-term safety profiles, pharmacokinetic characteristics, and strategies for tissue-specific targeting have not been fully elucidated. A considerable number of investigations use crude extracts with poorly defined chemical compositions, which greatly compromises methodological reproducibility. In addition, the bidirectional regulatory actions of HIF-1α are highly context-dependent, such that precise therapeutic windows must be defined to avoid off-target or adverse effects.

Accordingly, future investigations should focus on the following priorities: (1) development and application of standardized extracts and pure compounds accompanied by rigorous quality control procedures; (2) systematic pharmacokinetic evaluations and dose-optimization studies; (3) validation in large animal models and subsequent translation into controlled clinical trials; (4) design of targeted delivery systems to improve myocardial specificity; and (5) rational multi-target combination strategies that enhance therapeutic efficacy while reducing off-target risks.
